# Supplementary material for: Evaluation of the Presence of ASFV in Wolf Feces Collected from Areas in Poland with ASFV Persistence
Source: Viruses. 2021 Oct 14;13(10):2062. doi: 10.3390/v13102062 (PMC8541390; doi:10.3390/v13102062)
Supplement: Supplementary file 1 [file viruses-13-02062-s001.zip › viruses-1377592-supplementary.pdf]

viruses MDPI

Supplementary information for:

Evaluation of the Presence of ASFV in Wolf Feces Collected From Areas in Poland With ASFV Persistence

Maciej Szewczyk[1,*], Krzysztof Łepek[2,*], Sabina Nowak[3], Małgorzata Witek[1], Anna Bajcarzyk[2], Korneliusz Kurek[4], Przemysław Stachyra[5], Robert W. Mysłajek[3,✉], and Bogusław Szewczyk[2 ✉]

[1] Department of Vertebrate Ecology and Zoology, Faculty of Biology, University of Gdańsk, Wita Stwosza 59, 80-308 Gdańsk, Poland
[2] Department of Recombinant Vaccines, Intercollegiate Faculty of Biotechnology, University of Gdańsk, Abrahama 58, 80-307 Gdańsk, Poland
[3] Department of Ecology, Institute of Functional Biology and Ecology, Faculty of Biology, University of Warsaw, Biological and Chemical Research Centre, Żwirki i Wigury 101, 02-089 Warszawa, Poland
[4] Masurian Centre for Biodiversity, Research and Education, Faculty of Biology, University of Warsaw, Urwitałt 1, 11-730 Mikołajki, Poland
[5] Roztocze National Park, Plażowa 2, 22-470 Zwierzyniec, Poland

Supplementary Methods

Preparation of PCR-positive control and PCR optimization

A 315 bp DNA fragment (sequence below) corresponding to base pairs 1542-1857 of the ASFV *VP72* gene was commercially synthesized by Life Technologies. It contained introduced BamHI restriction sites (red) that flanked the 257 bp region (bolded) amplified by PPA-1/PPA-2 primers (Aguero et al. 2003)

5′-GCTGCCCATGGGGATCCTCTGGGACGTGCCCTGAATCGGAGCATCTT-GCCAG-GATGAATGACATGCACCCAATATATGATGGCCCACCATATCATGGAAAAAGTCTCCGTACTGGGGAATACCAAAGGTAAGCTTGTTTCCCAAGGTGGGGGTACCCG-TATGCGGGCG-TACTTTATTGTATTCAAACCCTACTGGAACATAAGGCTTAAAATGCGCATTAAAATGCACCAAATGTGTTTCTTCGATTTGACTCAAAGTGGGTTCGG-GATCGGGTTTCCCATAACTTTTGTTCACATGGATCCTGTTAGAGATCCT-3′

The synthesized fragment was cloned to pJET1.2/blunt vector (ThermoScientific) using T4 DNA ligase (ThermoScientific) and subsequently transformed into E. coli TOP10 cells. Transformed bacteria were selected on LB-agar plates supplemented with ampicillin. Single colonies were picked and cultured overnight in LB medium with ampicillin. Next, plasmid DNA was isolated using the Plasmid mini kit (A&A Biotechnology). To confirm the presence of insert, we checked isolated plasmids by BamHI digestion followed by agarose electrophoresis.

Prepared DNA construct was used to optimize PCR conditions and served as a positive control. Plasmid DNA (100 ng/uL) was serially diluted in 10^{-1} – 10^{-10} range and used as a template in PCR reaction with PPA-1/PPA-2 primers (Aguero et al. 2003, see main text). We tested the effect of number of PCR cycles on PCR specificity. For 40 cycles, we observed a PCR product of predicted size (257 bp) up to 10^{-7} dilution, but for 10^{-6} and 10^{-7} dilutions, some unspecific products were also detected. Thus, we selected 35 as an optimal number of cycles, as in these conditions, we observed clear single bands at 257 bp up to 10^{-5} template dilution, and unspecific products were not present at any dilution. In subsequent PCR experiments, we used 0.01 ng/uL plasmid DNA (i.e., 10^{-4} template dilution) as a PCR-positive control.

Isolation control experiment

E. coli TOP10 cells carrying plasmid containing fragment of ASFV VP72 gene were cultured overnight in LB medium supplemented with ampicillin and serially diluted (10-

^{2}, 10^{-4}, 10^{-6}) in medium. A fresh wolf scat (not preserved in ethanol) was divided into eight equal pieces (≈1x2 cm) and 50 μL of cell suspended in medium (undiluted or of indicated dilution) was added to each piece (two replicates per dilution). Samples were then incubated for 24 or 72 h in atmospheric conditions (temperature range ≈10–20 °C, no precipitation during first 24 h, but light rain between 24 and 72 h time points), followed by DNA isolation and PCR (as described in the main text).

Supplementary Tables

Table S1. List of analyzed wolf fecal samples.

scat_ID	individual	sample collection date	country	region	forest district	ASF zone	longitude	lattitude	collected by	prey item	figure	lane number
ROZ228	M1	2021-03-16	Poland	Roztocze National Park	Zwierzyniec	yes	23.1055	50.5519	S. Nowak, R. Mysłajek, P. Stachyra	wild boar	Fig. 2	9
ROZ229	M1	2021-03-25	Poland	Roztocze National Park	Roztocze National Park	yes	23.0406	50.6445	S. Nowak, R. Mysłajek, P. Stachyra	wild boar	Fig. 2	10
ROZ230	M1	2021-01-16	Poland	Roztocze National Park	Roztocze National Park	yes	23.1514	50.5246	S. Nowak, R. Mysłajek, P. Stachyra	wild boar	Fig. 2	11
ROZ231	M1	2021-02-21	Poland	Roztocze National Park	Roztocze National Park	yes	23.0612	50.6373	S. Nowak, R. Mysłajek, P. Stachyra	wild boar	Fig. 2	12
ROZ232	M1	2021-02-21	Poland	Roztocze National Park	Roztocze National Park	yes	23.0612	50.6353	S. Nowak, R. Mysłajek, P. Stachyra	wild boar	Fig. 2	13
ROZ233	M1	2021-04-27	Poland	Roztocze National Park	Roztocze National Park	yes	23.0611	50.6359	S. Nowak, R. Mysłajek, P. Stachyra	wild boar	Fig. 2	14
PP29		2020-04-19	Poland	Pisz Forest	Nowogród	yes	21.6405	53.3293	M. Szewczyk	wild boar	Fig. 2	1
PP35	M2	2021-01-11	Poland	Pisz Forest	Giżycko	yes	21.8590	53.8578	K. Kurek	wild boar	Fig. 2	2
PP38	M2	2021-01-11	Poland	Pisz Forest	Giżycko	yes	21.8184	53.8481	K. Kurek	wild boar	Fig. 2	3
PP60	M2	2021-02-11	Poland	Pisz Forest	Giżycko	yes	21.7703	53.8483	K. Kurek	wild boar	Fig. 2	4
PP61	M2	2021-02-14	Poland	Pisz Forest	Giżycko	yes	21.7596	53.8276	K. Kurek	wild boar	Fig. 2	5
PP63	M2	2021-02-14	Poland	Pisz Forest	Giżycko	yes	21.7532	53.8419	K. Kurek	wild boar	Fig. 2	6
PP64	M2	2021-02-17	Poland	Pisz Forest	Giżycko	yes	21.7520	53.8411	K. Kurek	wild boar	Fig. 2	7
PP65	M2	2021-02-14	Poland	Pisz Forest	Giżycko	yes	21.7652	53.8308	K. Kurek	wild boar	Fig. 2	8
LGRA04		2019-12-21	Poland	Bielsk Plain	Bielsk	yes	23.0679	52.8729	M. Szewczyk	wild boar	Fig. 2	15
LGRA06		2019-12-21	Poland	Bielsk Plain	Bielsk	yes	23.0172	52.7942	M. Szewczyk	wild boar	Fig. 2	16
LGRA08		2019-12-29	Poland	Bielsk Plain	Bielsk	yes	23.0894	52.8737	M. Szewczyk	wild boar	Fig. 2	17
PML02		2019-12-23	Poland	Mielnik Forest	Nurzec	yes	23.1361	52.3299	M. Szewczyk	wild boar	Fig. 2	18
PML04		2019-12-23	Poland	Mielnik Forest	Nurzec	yes	23.1967	52.3720	M. Szewczyk	wild boar	Fig. 2	19
PML05		2019-12-25	Poland	Mielnik Forest	Nurzec	yes	23.1189	52.6168	N. Niedźwiecka	wild boar	Fig. 2	20
BIA013		2018-07-15	Poland	Biała Forest	Sokołów	yes	22.3102	52.6314	M. Szewczyk	n/a	Fig. S2	1
BIA015		2019-05-26	Poland	Biała Forest	Ostrów Mazowiecka	yes	21.7512	52.7137	Barbara Czuba	n/a	Fig. S2	2
BIA016		2019-05-26	Poland	Biała Forest	Ostrów Mazowiecka	yes	21.7431	52.7134	Barbara Czuba	n/a	Fig. S2	3
BIA017		2019-12-05	Poland	Biała Forest	Wyszków	yes	21.5102	52.6862	A. Haidt	n/a	Fig. S2	4
BPN11		2018-03-30	Poland	Biebrza River Valley	Biebrza National Park	yes	22.5623	53.3717	M. Szewczyk	n/a	Fig. S2	5
BPN19		2018-02-13	Poland	Biebrza River Valley	Biebrza National Park	yes	22.7748	53.5880	K. Henel	n/a	Fig. S2	6
BPN23		2018-03-21	Poland	Biebrza River Valley	Biebrza National Park	yes	22.7622	53.6061	H. Ohwalińska	n/a	Fig. S2	7
LGRA05		2019-12-21	Poland	Bielsk Plain	Bielsk	yes	23.0889	52.8737	M. Szewczyk	n/a	Fig. S2	15
LGRA07		2019-12-25	Poland	Bielsk Plain	Bielsk	yes	23.0321	52.7845	N. Niedźwiecka, M. Szewczyk	n/a	Fig. S2	16
LNR136		2018-12-31	Poland	Napiwoda-Ramuki Fores	Przasnysz	yes	20.7124	53.2733	K. Stępniak	n/a	Fig. S2	28
LNR144		2019-02-17	Poland	Napiwoda-Ramuki Fores	Jedwabno	yes	20.6842	53.4377	K. Stępniak	n/a	Fig. S2	29
LNR146		2019-02-17	Poland	Napiwoda-Ramuki Fores	Jedwabno	yes	20.7224	53.4823	K. Stępniak	n/a	Fig. S2	30
LNR152		2019-02-17	Poland	Napiwoda-Ramuki Fores	Jedwabno	yes	20.7069	53.4367	K. Stępniak	n/a	Fig. S2	31
LNR161		2019-03-10	Poland	Napiwoda-Ramuki Fores	Jedwabno	yes	20.7708	53.4635	K. Stępniak	n/a	Fig. S2	32
LS10		2018-12-08	Poland	Spała Forest	Grójec	yes	20.6532	51.5905	I. Kwiatkowska	n/a	Fig. S2	8
PBi16		2018-03-10	Poland	Białowieża Forest	Białowieża National Par	yes	23.7167	52.6315	K. Stępniak	n/a	Fig. S2	13
PBi21		2018-03-10	Poland	Białowieża Forest	Białowieża National Par	yes	23.3390	52.5960	M. Szewczyk	n/a	Fig. S2	14
PK041		2018-12-27	Poland	Kampinos National Park	Kampinos National Park	yes	20.4422	52.3522	K. Stępniak, I. Kwiatkowska	n/a	Fig. S2	11
PK055		2019-02-14	Poland	Kampinos National Park	Kampinos National Park	yes	20.4328	52.3456	K. Stępniak	n/a	Fig. S2	12
PML03		2019-12-23	Poland	Mielnik Forest	Nurzec	yes	23.1971	52.3719	M. Szewczyk	n/a	Fig. S2	9
PML07		2019-12-29	Poland	Mielnik Forest	Nurzec	yes	23.1363	52.6109	M. Szewczyk	n/a	Fig. S2	10
POL05		2018-05-18	Poland	Polesie National Park	Parczew	yes	22.9600	51.5443	M. Kołodziejczyk	n/a	Fig. S2	26
POL20		2019-03-25	Poland	Polesie National Park	Polesie National Park	yes	23.2539	51.4652	Wołoszewicz	n/a	Fig. S2	27
ROZ105		2019-03-10	Poland	Roztocze National Park	PN Roztoczański	yes	23.0612	50.6329	R. Mysłajek	n/a	Fig. S2	17
ROZ136		2020-01-14	Poland	Roztocze National Park	Zwierzyniec	yes	22.8402	50.5987	S. Nowak, R. Mysłajek	n/a	Fig. S2	18
ROZ137		2020-01-14	Poland	Roztocze National Park	Zwierzyniec	yes	22.8403	50.5987	S. Nowak, R. Mysłajek	n/a	Fig. S2	19
ROZ153		2020-01-24	Poland	Roztocze National Park	PN Roztoczański	yes	23.0542	50.6105	R. Mysłajek, S. Nowak, M. Szewczyk	n/a	Fig. S2	20
SL037		2019-03-05	Poland	Solska Forest	Zwierzyniec	yes	22.8358	50.6035	R. Mysłajek, S. Nowak, P. Stachyra	n/a	Fig. S2	21
SL042		2019-02-14	Poland	Solska Forest	Zwierzyniec	yes	22.9359	50.5306	R. Mysłajek, S. Nowak, P. Stachyra	n/a	Fig. S2	22
SL046		2019-02-15	Poland	Solska Forest	Zwierzyniec	yes	23.0698	50.6841	R. Mysłajek, S. Nowak	n/a	Fig. S2	23
SL047		2019-03-02	Poland	Solska Forest	Józefów	yes	23.0585	50.3970	R. Mysłajek, S. Nowak, P. Stachyra	n/a	Fig. S2	24
SL060		2020-01-23	Poland	Solska Forest	Biłgoraj	yes	22.7606	50.5767	M. Szewczyk	n/a	Fig. S2	25
BD569		2018-03-10	Poland	Lower Silesian Forest	Przemków	no	15.7138	51.4593	R.Mysłajek, S. Nowak	n/a	n/a	n/a
BT318		2019-04-22	Poland	Tuchola Forest	Dąbrowa	no	18.5173	53.5982	M. Szewczyk	n/a	n/a	n/a
BT323		2019-04-28	Poland	Tuchola Forest	Dąbrowa	no	18.5434	53.5390	M. Szewczyk	n/a	n/a	n/a
BT332		2019-07-20	Poland	Tuchola Forest	Tuchola	no	18.0361	53.6477	M. Szewczyk	n/a	n/a	n/a
PN148		2018-08-20	Poland	Noteć Forest	Oborniki	no	16.7137	52.7266	R. Mysłajek, S. Nowak, P. Tomczak	n/a	n/a	n/a
PN155		2018-07-22	Poland	Noteć Forest	Karwin	no	15.7471	52.7944	P. Tomczak	n/a	n/a	n/a
PN166		2019-02-24	Poland	Noteć Forest	Sierakowice	no	16.1378	52.7316	Kasprzak	n/a	n/a	n/a
BD575		2018-03-11	Poland	Lower Silesian Forest	Świętoszów	no	15.5190	51.4964	R.Mysłajek, S. Nowak	n/a	n/a	n/a
BD577		2018-03-10	Poland	Lower Silesian Forest	Przemków	no	15.7157	51.4559	R.Mysłajek, S. Nowak	n/a	n/a	n/a
PB085		2019-02-09	Poland	Bydgoszcz Forest	Szubin	no	17.4401	53.0469	J. Napierała	n/a	n/a	n/a

Table S2. List of analyzed wild boar tissue samples.

tissue_ID	species	individual	country	region	sample collection date	approximate time of death	longitude	lattitude	collected by	figure	lane number	ASF detected
Dz01	Sus scrofa		Poland	Roztocze National Park	16.03.2021		23.1219	50.5536	P. Stachyra, S. Nowak, R. Mysłajek	Fig. 2	21	yes
Dz02	Sus scrofa		Poland	Roztocze National Park	16.03.2021		23.1215	50.5537	P. Stachyra, S. Nowak, R. Mysłajek	Fig. 2	22	no
Dz03	Sus scrofa		Poland	Roztocze National Park	25.03.2021		23.0444	50.6392	P. Stachyra, S. Nowak, R. Mysłajek	Fig. 2	23	yes
Dz04	Sus scrofa		Poland	Roztocze National Park	25.03.2021		23.0443	50.6390	P. Stachyra, S. Nowak, R. Mysłajek	Fig. 2	24	yes
Dz05	Sus scrofa		Poland	Roztocze National Park	25.03.2021		23.0437	50.6361	P. Stachyra, S. Nowak, R. Mysłajek	Fig. 2	25	yes
Dz06	Sus scrofa	ad male	Poland	Pisz Forest	28.03.2021	≈01.02.2021	21.7696	53.8484	K. Kurek	Fig. 2	26	yes
Dz07	Sus scrofa	ad	Poland	Pisz Forest	28.03.2021	26.03.2021	21.7616	53.8441	K. Kurek	Fig. 2	27	yes
Dz08	Sus scrofa	juv/subad	Poland	Pisz Forest	28.03.2021	27.03.2021	21.7591	53.8377	K. Kurek	Fig. 2	28	yes
Dz09	Sus scrofa	juv/subad	Poland	Pisz Forest	28.03.2021	27.03.2021	21.7592	53.8376	K. Kurek	Fig. 2	29	yes

Supplementary Figures

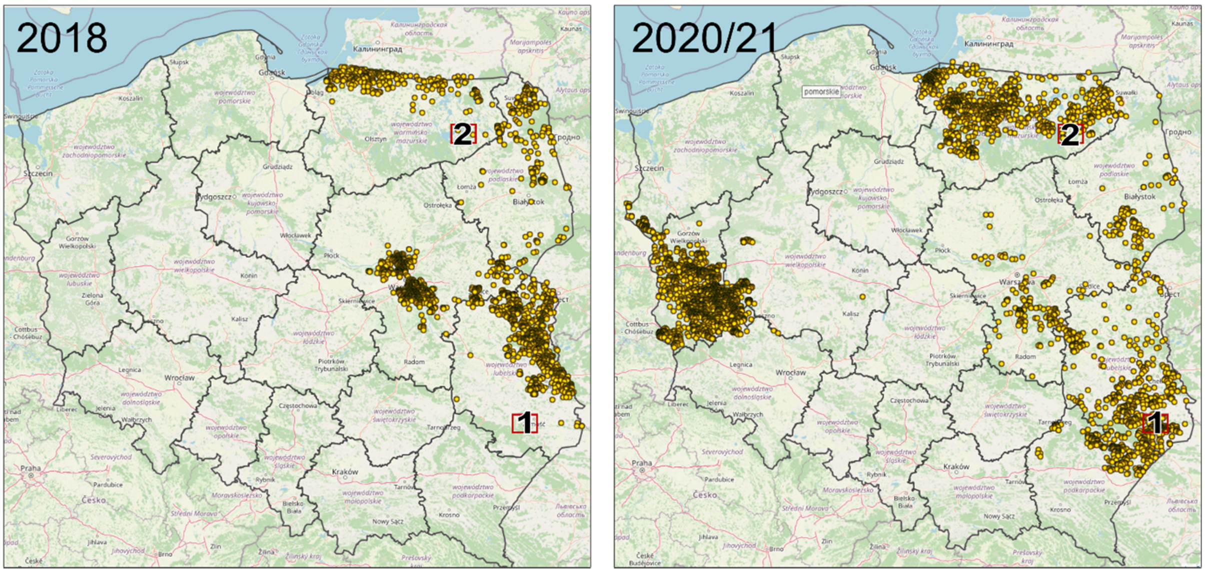

Figure S1. ASF occurrence among wild boar population in Poland at the beginning (2018) and end (2021) of our study. Areas where samples were collected during telemetric studies on wolves are indicated with red rectangles (1—wolf M1, 2—wolf M2). Source: National Veterinary Research Institute, Puławy, Poland (https://bip.wetgiw.gov.pl/asf/mapa/).

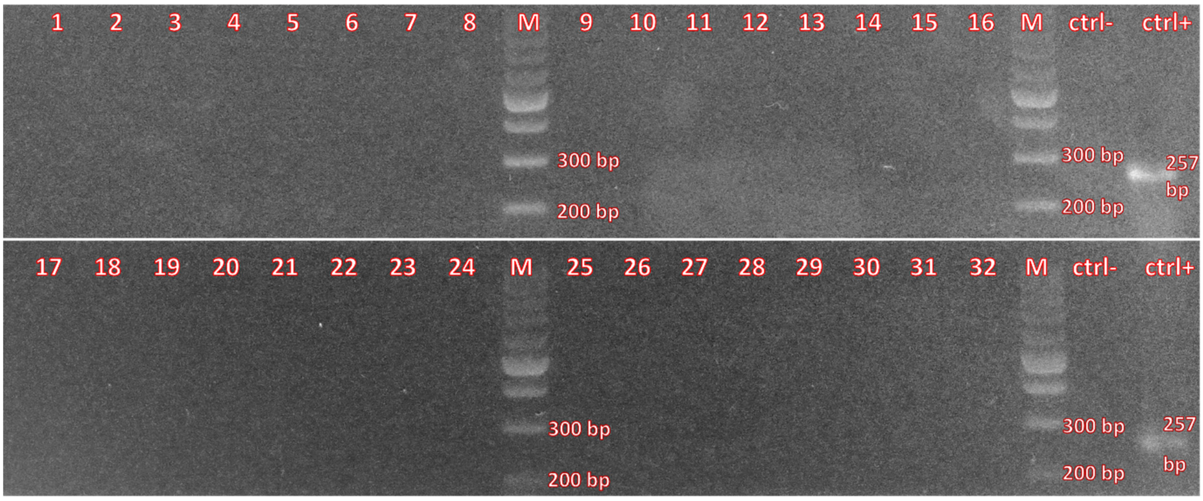

Figure S2. ASFV genetic material was not detected in 32 eastern Polish wolf fecal samples collected in 2018-2019. Ctrl- represents PCR-negative control (elution buffer from DNeasy PowerFaecal Pro Kit used as template), ctrl+ represents PCR-positive control (plasmid DNA containing the amplified fragment of ASFV VP72 gene used as template). For lane numbers and detailed information on samples, see Table S1.

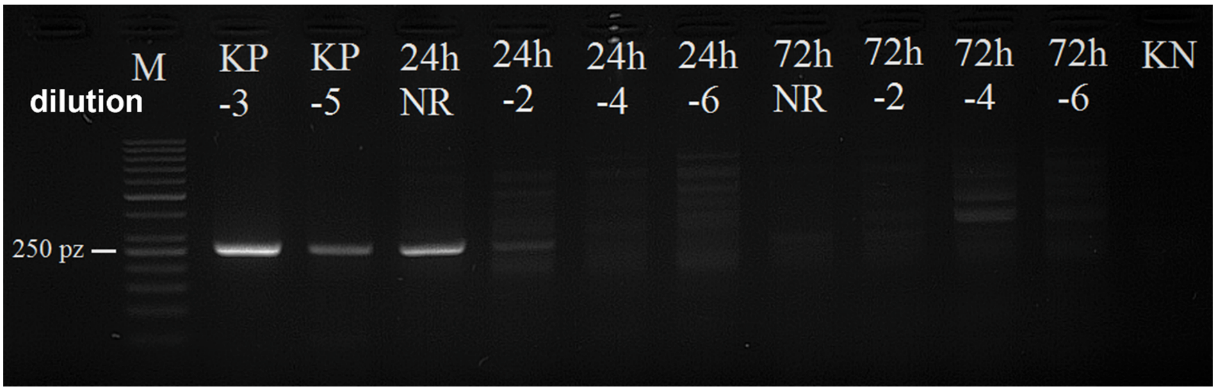

Figure S3. Isolation control experiment shows that amplified fragment of ASFV genetic material could be isolated from fecal samples. For details, see the Supplementary Methods. KP—positive control, KN—negative control, NR—undiluted; numbers indicate template dilution (-3—10^{-3}, -5—10^{-5}, etc.).

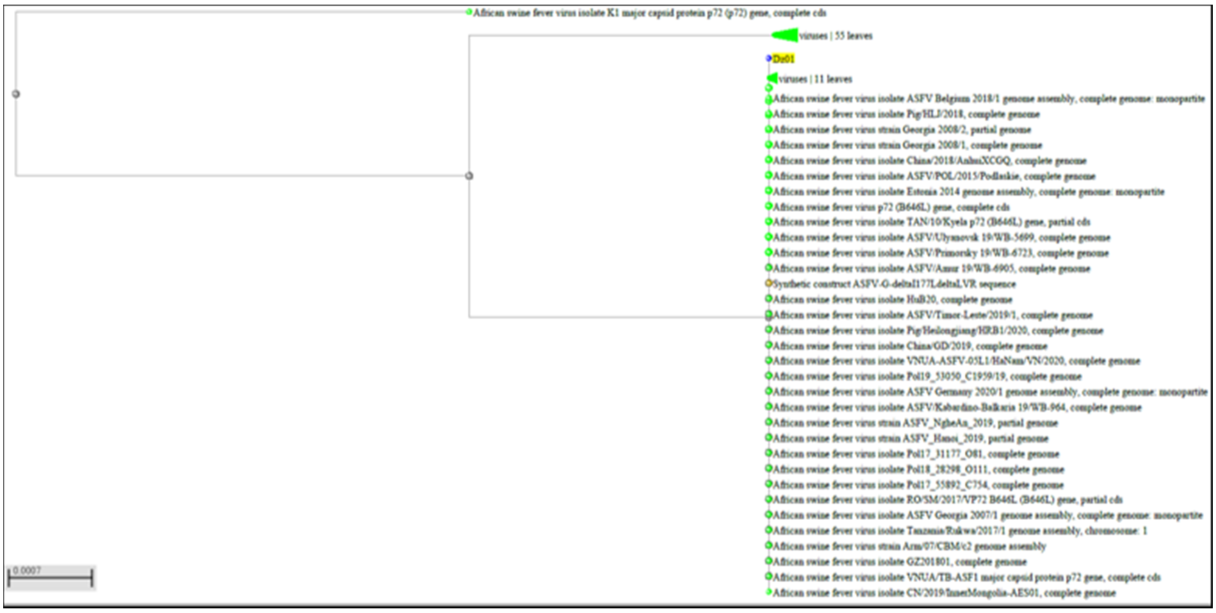

Figure S4. Phylogenetic relationship between sequence from ASFV-positive wild boar tissue samples and nearest hits from the NCBI database. PCR products from all 8 positive samples had the same sequence, so only one sample (Dz01) is shown in the phylogenetic tree. Sequences on the same branch have 100% identity.
